# Supplementary material for: Mathematical modeling of the impact of cytokine response of acute myeloid leukemia cells on patient prognosis
Source: Sci Rep. 2018 Feb 12;8:2809. doi: 10.1038/s41598-018-21115-4 (PMC5809606; doi:10.1038/s41598-018-21115-4)
Supplement: Supplementary file 1 — Supplementary Information [file 41598_2018_21115_MOESM1_ESM.pdf]

Supplementary Methods: Mathematical modeling of the impact of cytokine response of acute myeloid leukemia cells on patient prognosis.

Thomas Stiehl[1], Anthony D Ho[2], and Anna Marciniak-Czochra[1]

[1]Institute of Applied Mathematics, Interdisciplinary Center of Scientific Computing and BIOQUANT Center, Heidelberg University, Germany
[2]Department of Medicine V, Heidelberg University Hospital, Germany

Contents

1 Model Derivations **2**
1.1 Model of hematopoiesis . . . 2
1.2 Healthy steady state . . . 5
1.3 Model of a cytokine-dependent AML (Model 1) . . . 6
1.4 Model of a cytokine-independent AML (Model 2) . . . 7

2 Model Analysis **10**
2.1 Biological interpretation of steady states . . . 10
2.2 Linearized stability analysis for $n = m = 2$ . . . 10
2.3 Leukemic stem cell properties . . . 16
2.4 Criteria for instability of steady states . . . 17
2.5 Comparison of Models 1 and 2 . . . 18
2.5.1 Steady states . . . 18
2.5.2 Leukemic stem cell properties . . . 18
2.5.3 Stability results . . . 19

3 Simulation and Fitting **19**
3.1 Simulation of cytokine administration . . . 19
3.2 Fitting to data of 41 patients . . . 20
3.3 Fitting to data of cytokine administration . . . 22

3.4 Simulations shown in Figure 3 22
3.5 Simulations shown in Figure 6 23

1 Model Derivations

1.1 Model of hematopoiesis

Compartmental structure and model parameters

We consider one hematopoietic lineage consisting of an ordered sequence of n maturation steps (compartments)[1,2]. The first compartment describes the population of hematopoietic stem cells (HSC) and the nth compartment stands for the population of post-mitotic mature cells, e.g., granulocytes. We denote by $c_i(t)$ $(i = 1, \ldots, n)$ the cell counts in the hematopoietic compartment i at time t. Cells in compartment i are characterized by the following properties.

- **Proliferation rate** $p_i^c(t)$ in compartment i at time t, describing how often a cell divides per unit of time. We assume that cells of the most mature compartment are post-mitotic, i.e., $p_n^c(t) \equiv 0$.

- **Fraction of self-renewal** $a_i^c(t)$ in compartment i at time t, i.e., the fraction of progeny cells returning to the compartment occupied by the parent cells that gave rise to them (process referred to as self-renewal). The fraction $1 - a_i^c(t)$ of progeny cells moves on to the compartment $i + 1$ (process referred to as differentiation).

- **Death rate** d_i^c in compartment i, describing how many cells die per unit of time. For simplicity, death rates are assumed to be zero or constant in time. We further assume $d_n^c > 0$[1].

Definition 1 (Effective growth rate)**.** *Consider an arbitrary mitotic cell compartment. Let $p(t)$ be the proliferation rate at time t, $a(t)$ the fraction of self-renewal and $d(t)$ the death rate. Then the effective growth rate of this cell compartment is defined as $(2a(t) - 1)p(t) - d(t)$. It describes the number of cells of the compartment that originate due to cell divisions and self-renewal subtracted by cell death.*

Control feedback

Regulation of the granulocytes production is modeled by a negative feedback loop between the level of granulocytes and the level of cytokines which,

in turn, control the differentiation process. The formula for the signal concentration is given by a Hill-function $s(t) = \frac{1}{1+kc_n(t)} \in (0,1]$, where k is a positive constant[3,4]. It has been derived using a quasi steady-state approximation of G-CSF dynamics[3,4].

On the basis of our earlier work and on compatibility with clinical data[3,5,6], we assume feedback inhibition of the fraction of self-renewal by mature cells. The fraction of self-renewal of the healthy cells is assumed to be given by $a_i^c(t) \equiv a_i^c s(t)$. This assumption results in a good qualitative and quantitative agreement with clinical data during hematopoietic stress[3,5–7] and in leukemias[8–10]. The opposite assumption, i.e., increased differentiation in case of reduced mature blood cell counts, cannot reproduce clinical observations after bone marrow transplantation or in case of external cytokine administration[6].

We neglect the impact of cytokines on the proliferation rates. Our previous quantitative modeling works, see references[3,5–7], have shown that additional regulation of proliferation rates has little impact on model dynamics. A system with a regulation of proliferation rates but constant self-renewal /differentiation is insufficient to explain hematopoietic reconstitution[3,6,7]. The simulations depicted in Supplemental Figure 1 show that in our leukemia models additional regulation of proliferation rates has little impact on the blast dynamics.

Model equations

The flux to division in compartment $i < n$ at time t equals $p_i^c(t)c_i(t)$. During division a parent cell is replaced by two progeny cells. The outflux from division at time t equals $2p_i^c(t)c_i(t)$, of which the fraction $2a_i^c(t)p_i^c(t)c_i(t)$ stays in compartment i. The fraction $2\big(1-a_i^c(t)\big)p_i^c(t)c_i(t)$ moves to compartment $i+1$. This results in the following model of healthy hematopoiesis, which has been validated and applied to clinical data[3,5,6].

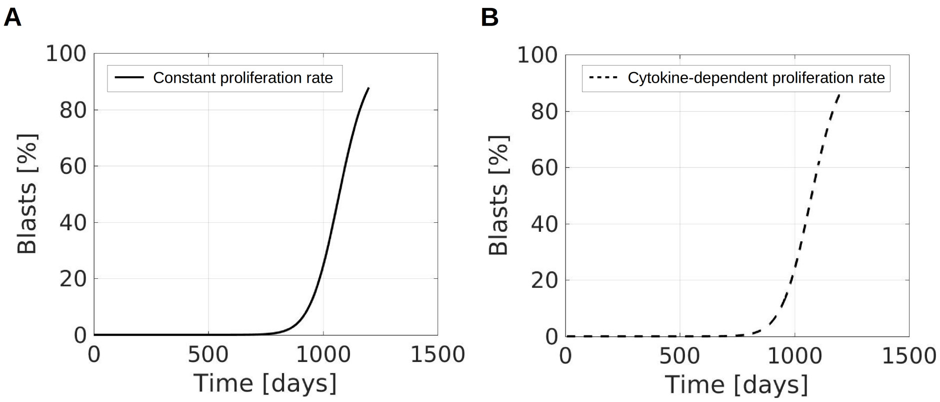

Supplemental Figure 1: Impact of cytokine regulated proliferation rates. (A) Simulation of the model of cytokine-dependent AML as given by the system of equations (2). In this model the impact of cytokines on proliferation rates is neglected and cytokines regulate the fraction of differentiating versus self-renewing cells. (B) Simulation of a model of cytokine-dependent AML including cytokine-dependent proliferation rates. In this version of the model cytokines regulate both, proliferation rate and self-renewal versus differentiation. The corresponding system of equations is obtained from system (2) by replacing p_i^c by $p_i^c s(t)$ and p_i^l by $p_i^l s(t)$. Proliferation rates in the healthy steady state in panel (B) are identical to the constant proliferation rates in panel (A). Blast dynamics are very similar in both panels. Parameters: (A) $a_1^c = 0.87$, $p_1^c = 0.45$, $a_1^l = 0.9$, $p_1^l = 0.6$, $k^c = 1.85 \cdot 10^{-9}$, $d_2^c = 2.3$, $d_2^l = 0.5$, $k^c = k^l$. (B) $p_1^c = 0.45/\bar{s}$, $p_1^l = 0.6/\bar{s}$, where $\bar{s} = \frac{1}{2a_1^c}$ is the cytokine level in healthy steady state. The remainder is as in Panel (A).

$$
\begin{aligned}
\frac{d}{dt}c_1(t) &= \big(2a_1^c s(t)-1\big)p_1^c c_1(t) - d_1^c c_1(t),\\
\frac{d}{dt}c_i(t) &= 2\big(1-a_{i-1}^c s(t)\big)p_{i-1}^c c_{i-1}(t) + \big(2a_i^c s(t)-1\big)p_i^c(t)c_i(t)\\
&\quad -d_i^c c_i(t), \quad 1<i<n,\\
\frac{d}{dt}c_n(t) &= 2\big(1-a_{n-1}^c s(t)\big)p_{n-1}^c c_{n-1}(t) - d_n^c c_n(t),\\
s(t) &= \frac{1}{1+kc_n(t)}, \qquad (1)
\end{aligned}
$$

with initial data given by $c_1(0) > 0, c_2(0) \geq 0, \cdots, c_n(0) \geq 0$ and the parameters satisfying the following assumptions.

Assumptions 2. *(i)* $a_i^c \in (0,1)$ *for* $1 \leq i \leq n-1$,

(ii) $p_i^c > 0$ *for* $1 \leq i \leq n-1$,

(iii) $d_i^c \geq 0$ *for* $1 \leq i \leq n-1$, $d_n^c > 0$,

(iv) $k > 0$.

1.2 Healthy steady state

We cite the following result stating existence and uniqueness of a positive equilibrium of the healthy cells[4].

Proposition 3 (Positive Steady States)**.** *Let Assumptions 2 be fulfilled. Then system (1) has a unique positive steady state* $\bar{c}_1, ..., \bar{c}_n$, *if and only if the following conditions are satisfied:*

(1) $(2a_1 - 1)p_1 > d_1$

(2) $2a_1p_1(d_i + p_i) - 2a_ip_i(d_1 + p_1) > 0, \text{ for } i = 2, \ldots, n-1.$

The steady state is given by

$$\bar{c}_l = \bar{c}_n \Pi_{l+1}^n \Theta_i, \text{ for } l = 1, \ldots, n,$$

where

$\bar{c}_n = \frac{1}{k}(\frac{2a_1p_1}{d_1+p_1} - 1)$,

$\Theta_i := \frac{d_i+p_i-2a_ip_i\bar{s}}{2(1-a_{i-1}\bar{s})p_{i-1}} > 0, \text{ for } i = 2, \ldots, n-1,$

$\Theta_n := \frac{d_n}{2(1-a_{n-1}\bar{s})p_{n-1}} > 0$, *and*

$\bar{s} := \frac{d_1+p_1}{2a_1p_1}$.

1.3 Model of a cytokine-dependent AML (Model 1)

We denote by $l_i(t)$ the density of leukemic cells of type i at time t. Proliferation rate, fraction of self-renewal and death rate of cells in leukemic compartment i at time t are denoted as $p_i^l(t)$, $a_i^l(t)$ and $d_i^l(t)$. A scheme of the model is presented in Figure 1A.

Control feedback

In Model 1, it is assumed that leukemic cells depend on the same cytokines as healthy hematopoietic cells. Since hematopoietic and leukemic cells express G-CSF receptors[16,17] and degrade G-CSF by receptor-mediated endocytosis, the two cell lineages interact through competition for the cytokine. To model this competition we assume degradation of the signal s by the most mature leukemic cells and by granulocytes. Using a quasi-steady state assumption, this results in: $s(t) := \frac{1}{1+k^c c_n(t)+k^l l_m(t)}$ or $s(t) := \frac{1}{1+k^c c_n(t)+\sum_{i=1}^m k_i^l l_i(t)}$[18]. The latter expression takes into account signal degradation by all leukemic cell types. Here k^c, k^l and k_i^l are positive constants. As for hematopoietic cells, we assume that the cytokine level s controls the self-renewal of leukemic cells.

Model equations

We obtain the following system of model equations.

$$
\begin{aligned}
\frac{d}{dt}c_1(t) &= \big(2a_1^c s(t)-1\big)p_1^c c_1(t) - d_1^c c_1(t),\\
\frac{d}{dt}c_i(t) &= 2\big(1-a_{i-1}^c s(t)\big)p_{i-1}^c c_{i-1}(t) + \big(2a_i^c s(t)-1\big)p_i^c(t)c_i(t)\\
&\quad -d_i^c c_i(t), \quad 1<i<n,\\
\frac{d}{dt}c_n(t) &= 2\big(1-a_{n-1}^c s(t)\big)p_{n-1}^c c_{n-1}(t) - d_n^c c_n(t),\\
\frac{d}{dt}l_1(t) &= \big(2a_1^l s(t)-1\big)p_1^l l_1(t) - d_1^l l_1(t),\\
\frac{d}{dt}l_i(t) &= 2\big(1-a_{i-1}^l s(t)\big)p_{i-1}^l l_{i-1}(t) + \big(2a_i^l s(t)-1\big)p_i^l(t)l_i(t)\\
&\quad -d_i^l l_i(t), \quad 1<i<m,\\
\frac{d}{dt}l_m(t) &= 2\big(1-a_{m-1}^l s(t)\big)p_{m-1}^l l_{m-1}(t) - d_m^l l_m(t),\\
s(t) &= \frac{1}{1+k^c c_n(t)+k^l l_m(t)},
\end{aligned}
\tag{2}
$$

with given non-negative initial data $c_1(0),\ \cdots,\ c_n(0), l_1(0),\ \cdots,\ l_m(0)$.

Assumptions 4. *Following biological interpretation, we make the following assumptions on parameters:*

(i) $p_i^c > 0$ *for* $1 \le i \le n-1$, $p_i^l > 0$ *for* $1 \le i \le m-1$,

(ii) $a_i^c \in (0,1)$ *for* $1 \le i \le n-1$, $a_i^l \in (0,1)$ *for* $1 \le i \le m-1$,

(iii) $d_i^c \ge 0$ *for* $1 \le i \le n-1$, $d_i^l \ge 0$ *for* $1 \le i \le m-1$,

(iv) $d_n^c > 0$, $d_m^l > 0$,

(v) $k^c > 0$, $k^l > 0$,

(vi) $c_1(0) > 0$, $c_i(0) \ge 0$ *for* $2 \le i \le n$, $l_1(0) > 0$, $l_i(0) \ge 0$ *for* $2 \le i \le m$.

1.4 Model of a cytokine-independent AML (Model 2)

We use the same notation as in Model 1. The model is illustrated in Figure 1B.

Control feedback

We model the space competition by introducing a death rate that increases with the number of cells in bone marrow and acts on all cell types residing in bone marrow. This rate is described by a function $d_c : \mathbb{R}_0^+ \to \mathbb{R}_0^+$, where $\mathbb{R}_0^+ := \{x \in \mathbb{R} \mid x \geq 0\}$. We assume that d_c is a non-negative increasing, locally Lipschitz continuous function with $\lim_{x\to\infty} d_c(x) = \infty$. We further assume that under healthy conditions there exists no cell death due to space competition. This assumption is in line with bone marrow histology[19]. Therefore, we assume that $d_c(x) = 0$, if the total cell count is below a certain threshold $\hat{x}$ and that it is strictly monotonically increasing otherwise. The threshold is greater or equal than the healthy steady state marrow cell count. The healthy steady state marrow cell count is given by $\sum_{i=1}^{n-1} \bar{c}_i$, where $\bar{c}_1, \ldots, \bar{c}_n$ is the unique positive equilibrium of system (1).

For simplicity, we assume that all leukemic cells, except those of the last stage, stay in bone marrow. Cells of the last stage can either leave marrow or not. The number of leukemic cells exiting bone marrow is highly variable among individuals and only partially dependent on the leukemia sub-type[20–22]. We obtain the following system of ODEs.

Model equations

$$
\begin{aligned}
\frac{d}{dt}c_1(t) &= (2a_1^c s(t) - 1)p_1 c_1(t) - d_1^c c_1(t) - d_c(t)c_1(t)\\
\frac{d}{dt}c_2(t) &= 2(1 - a_1^c s(t))p_1^c c_1(t) + (2a_2^c s(t) - 1)p_2 c_2(t) - d_2^c c_2(t) - d_c(t)c_2(t)\\
&\vdots\\
\frac{d}{dt}c_i(t) &= 2(1 - a_{i-1}^c s(t))p_{i-1}^c c_{i-1}(t) + (2a_i^c s(t) - 1)p_i c_i(t) - d_i^c c_i(t) - d_c(t)c_i(t)\\
&\vdots\\
\frac{d}{dt}c_{n-1}(t) &= 2(1 - a_{n-2}^c s(t))p_{n-2}^c c_{n-2}(t) + (2a_{n-1}^c s(t) - 1)p_{n-1} c_{n-1}(t) - d_{n-1}^c c_{n-1}(t)\\
&\quad - d_c(t)c_{n-1}(t)\\
\frac{d}{dt}c_n(t) &= 2(1 - a_{n-1}^c s(t))p_{n-1}^c c_{n-1}(t) - d_n c_n(t)
\end{aligned}
\tag{3}
$$

$$\begin{aligned}
\frac{d}{dt}l_1(t) &= (2a_1^l - 1)p_1^l l_1(t) - d_1^l l_1(t) - d_c(t)l_1(t) \\
\frac{d}{dt}l_2(t) &= 2(1 - a_1^l)p_1^l l_1(t) + (2a_2^l - 1)p_2^l l_2(t) - d_2^l l_2(t) - d_c(t)l_2(t) \\
&\vdots \\
\frac{d}{dt}l_i(t) &= 2(1 - a_{i-1}^l)p_{i-1}^l l_{i-1}(t) + (2a_i^l - 1)p_i^l l_i(t) - d_i^l l_i(t) - d_c(t)l_i(t) \\
&\vdots \\
\frac{d}{dt}l_{m-1}(t) &= 2(1 - a_{m-2}^l)p_{m-2}^l l_{m-2}(t) + (2a_{m-1}^l - 1)p_{m-1}^l l_{m-1}(t) - d_{m-1}^l l_{m-1}(t) \\
&\quad - d_c(t)l_{m-1}(t) \\
\frac{d}{dt}l_m(t) &= 2(1 - a_{m-1}^l)p_{m-1}^l l_{m-1}(t) - d_m^l l_m(t) - d_c(t)l_m(t)\chi
\end{aligned} \tag{4}$$

$$\begin{aligned}
s(t) &= \frac{1}{1 + kc_n(t)} \\
d_c(t) &\equiv \hat{d}_c\left(\sum_{i=1}^{n-1} c_i(t) + \sum_{i=1}^{m-1} l_i(t) + \chi l_m(t)\right)
\end{aligned} \tag{5}$$

and $\chi \in [0,1]$ and given non-negative initial data $c_1(0),\ \ldots,\ c_n(0),\ l_1(0),\ \ldots,\ l_m(0)$.

Remark 5. *If $\chi = 0$, all leukemic cells of the last stage leave bone marrow, if $\chi = 1$ all leukemic cells remain in bone marrow.*

Assumptions 6. *Motivated by their biological meaning, we make the following assumptions:*

(i) $a_i^l \in (0,1) \subset \mathbb{R}$,

(ii) $p_i^l > 0$, *for* $1 \le i \le m-1$,

(iii) $d_i^l \ge 0$, *for* $1 \le i \le m-1$, $d_m^l > 0$,

(iv) $\hat{d}_c:\ \mathbb{R} \to \mathbb{R},\ \ x \mapsto \begin{cases} 0,\ x < \hat{x} \\ g(x),\ x \ge \hat{x} \end{cases}$,

$g : \mathbb{R}_0^+ \to \mathbb{R}_0^+$ *is locally Lipschitz continuous, strictly monotonically*

increasing with $g(\hat{x}) = 0$, $\lim_{x\to\infty} g(x) = \infty$ *and* $\hat{x} > \sum_{i=1}^{n} \bar{c}_i$. *By* $\bar{c}_i$ *we denote the unique positive steady state values of* c_i *in system* (1).

(v) $c_1(0) > 0, c_i(0) \geq 0$ *for* $2 \geq i \geq n$, $l_1(0) > 0$, $l_j(0) \geq 0$ *for* $2 \geq j \geq m$.

2 Model Analysis

2.1 Biological interpretation of steady states

Steady state values of c_i and l_i are denoted as $\bar{c}_i$ and $\bar{l}_i$, respectively. Similar as in[18], we consider three types of steady states:

Definition 7 (Healthy steady state). *A steady state satisfying* $\bar{c}_i > 0$ *for* $i = 1, \cdots, n$ *and* $\bar{l}_k = 0$, *for* $k = 1, \ldots, m$ *is referred to as a healthy steady state.*

Definition 8 (Purely leukemic steady state). *A steady state satisfying* $\bar{c}_i = 0$, *for* $i = 1, \ldots, n$ *and* $\bar{l}_k > 0$, *for a* $k \in \{1, \cdots, m\}$ *is referred to as a purely leukemic steady state.*

Remark 9. *A purely leukemic steady state is an abstract reference that is incompatible with survival.*

Definition 10 (Mixed steady state). *Steady states, where* $\bar{c}_i > 0$ *for at least one* $i \in \{1, \cdots, n\}$ *and* $\bar{l}_j > 0$ *for at least one* $j \in \{1, \cdots, m\}$, *are referred to as mixed steady states.*

Remark 11 (Healthy steady state). *Assumption 6 (iv) implies that for the unique positive steady state* $(\bar{c}_1, \cdots, \bar{c}_n)$ *of model* (1), $(\bar{c}_1, \cdots, \bar{c}_n, 0, \cdots, 0)$ *is a healthy steady state of the system* (3) - (5).

We now focus on the case $n = m = 2$. We come back to the general case later.

2.2 Linearized stability analysis for $n = m = 2$

To obtain further insights into the dynamics of Model 2, we provide a systematic linear stability analysis for the case $n = m = 2$. This is the minimal system describing interaction of healthy and leukemic cells. Numerical simulations imply similarity for systems with more compartments for wide parameter ranges. The linear stability analysis for Model 1 is described in[18].

For $n = m = 2$ Assumptions 6 reduce to:

Assumptions 12. *(i)* $d_2^l > 0$, $d_2^c > 0$, $d_1^l \geq 0$, $d_1^c \geq 0$, $\chi \in [0,1]$, $p_1^c > 0$, $p_1^l > 0$.

(ii) $0 < (2a_1^c - 1)p_1^c - d_1^c$,

(iii) $0 < (2a_1^l - 1)p_1^l - d_1^l$.

For $n = m = 2$ Model 2 has the following form:

$$\begin{aligned} \frac{d}{dt}c_1 &= (2a_1^c s - 1)p_1^c c_1 - d_c(c_1 + l_1 + \chi l_2)c_1 - d_1^c c_1 \\ \frac{d}{dt}c_2 &= 2(1 - a_1^c s)p_1^c c_1 - d_2^c c_2 \\ s &= \frac{1}{1 + kc_2} \end{aligned} \tag{6}$$

$$\begin{aligned} \frac{d}{dt}l_1 &= (2a_1^l - 1)p_1^l l_1 - d_c(c_1 + l_1 + \chi l_2)l_1 - d_1^l l_1 \\ \frac{d}{dt}l_2 &= 2(1 - a_1^l)p_1^l l_1 - d_2^l l_2 - \chi d_c(c_1 + l_1 + \chi l_2)l_2 \end{aligned} \tag{7}$$

with

$$c_1(0) = c_1^0 > 0,\ c_2(0) = c_2^0 \geq 0,\ l_1(0) = l_1^0 > 0,\ l_2(0) = l_2^0 \geq 0. \tag{8}$$

and d_c as defined in Assumption 6 (iv) with $\hat{x} > \bar{c}_1$, where $\bar{c}_1$ denotes the value of c_1 in the unique healthy steady state.

The following proposition classifies the steady states of system (6)-(8).

Proposition 13 (Steady states)**.** *Let Assumptions 12 be fulfilled. Then the following assertions hold.*

(i) System (6)-(8) *possesses a unique healthy steady state* $(\bar{c}_1,\ \bar{c}_2,\ 0,\ 0)$ *with* $\bar{c}_2 = \frac{1}{k}\left(\frac{2a_1^c p_1^c}{d_1^c + p_1^c} - 1\right)$ *and* $\bar{c}_1 = \frac{d_2^c}{p_1^c - d_1^c}\bar{c}_2$.

(ii) System (6)-(8) *possesses a unique purely leukemic steady state* $(0,\ 0,\ ,\bar{l}_1,\ \bar{l}_2)$. *Define* $\bar{x}$ *by* $d_c(\bar{x}) = (2a_1^l - 1)p_1^l - d_1^l$. *Then* $\bar{l}_1 = \frac{\bar{x}}{1+\alpha}$, $\bar{l}_2 = \alpha\bar{l}_1$, $\alpha = \frac{2(1-a_1^l)p_1^l}{d_2^l + \chi\left((2a_1^l - 1)p_1^l - d_1^l\right)}$.

(iii) If and only if $(2a_1^l - 1)p_1^l - d_1^l < (2a_1^c - 1)p_1^c - d_1^c$ *system* (6)-(8) *possesses a unique mixed steady state* $(\bar{c}_1,\ \bar{c}_2,\ \bar{l}_1,\ \bar{l}_2)$*. Define* $\bar{x}$ *by* $d_c(\bar{x}) = (2a_1^l - 1)p_1^l - d_1^l$*. Then* $\bar{c}_2 = \frac{1}{k}\left(\frac{2a_1^c p_1^c}{(2a_1^l-1)p_1^l - d_1^l + d_1^c + p_1^c} - 1\right)$, $\bar{c}_1 = \frac{d_2^c}{p_1^c - d_1^c - \left((2a_1^l-1)p_1^l - d_1^l\right)}\bar{c}_2$, $\bar{l}_1 = \frac{\bar{x}-\bar{c}_1}{1+\alpha}$, $\bar{l}_2 = \alpha\bar{l}_1$, $\alpha = \frac{2(1-a_1^l)p_1^l}{d_2^l + \chi\left((2a_1^l-1)p_1^l - d_1^l\right)}$.

Proof. (i) follows from Assumption 6 and a direct calculation. (ii) follows from $(2a_1^l - 1)p_1^l - d_1^l > 0$ and strict monotonicity of d_c for $x > \hat{x}$.

(iii) $\frac{d}{dt}l_1 = 0$ requires $\bar{d}_c = (2a_1^l - 1)p_1^l - d_1^l$, where $\bar{d}_c$ denotes the steady state value of d_c. If $(2a_1^l - 1)p_1^l - d_1^l > (2a_1^c - 1)p_1^c - d_1^c$, then $\frac{d}{dt}c_1 = 0$ implies $\bar{c}_1 = 0$ and consequently $\bar{c}_2 = 0$. If $(2a_1^l - 1)p_1^l - d_1^l = (2a_1^c - 1)p_1^c - d_1^c$, then either $\bar{c}_1 = 0$ or $\bar{s} = 1$. The latter implies $\bar{c}_2 = 0$ and thus also $\bar{c}_1 = 0$. Therefore, the condition $(2a_1^l - 1)p_1^l - d_1^l < (2a_1^c - 1)p_1^c - d_1^c$ is necessary for existence of a mixed steady state. Denote by $\bar{s} = \frac{\bar{d} + d_1^c + p_1^c}{2a_1^c p_1^c}$ the value of s in the mixed steady state and by $\bar{s}^H = \frac{d_1^c + p_1^c}{2a_1^c p_1^c}$ the value of s in the healthy steady state. It holds $\bar{s}^H < \bar{s}$. The steady state condition implies $\bar{c}_1 = \frac{d_2^c}{2p_1^c k}\frac{1-\bar{s}}{\bar{s}(1-a_1^c\bar{s})}$. The value of $\bar{c}_1$ is decreasing for increasing $\bar{s} \in (0,1]$. Therefore, the steady state value of c_1 in the mixed steady state is smaller than in the healthy steady state. Due to the definition of d_c it follows that $\bar{x}$ is larger that the value of c_1 in the healthy steady state, therefore $\bar{x} - \bar{c}_1 > 0$, where $\bar{c}_1$ denotes the value of c_1 in the mixed steady state. The remainder is a direct calculation using $\bar{x} = \bar{c}_1 + \bar{l}_1 + \chi\bar{l}_2$. □

For simplicity of calculations we make from now on the following assumptions:

Assumptions 14. *(i)* $d_1^l = d_1^c = 0$,

(ii) $\chi = 0$.

The following Remarks and Propositions systematically investigate linear stability of all possible steady states of system (6)-(8).

Remark 15 (Stability of the healthy equilirium)**.** *It follows directly from linearization that for* $(2a_1^l - 1)p_1^l - d_1^l < 0$ *the healthy steady state is linearly stable.*

Proposition 16 (Case $[2a_1^c - 1]p_1^c < [2a_1^l - 1]p_1^l$)**.** *Consider system* (6)-(8)*. Let Assumptions 12 and 14 hold. Let* $(2a_1^c - 1)p_1^c < (2a_1^l - 1)p_1^l$*. Then,*

(i) A unique purely hematopoietic and a unique purely leukemic steady state are the only nontrivial non-negative steady states of the system.

(ii) The purely leukemic steady state is linearly asymptotically stable. The purely hematopoietic steady state is unstable.

Proof. of Proposition 16

(i) follows from Proposition 13.

(ii) We denote the steady state value of d_c as $\bar{d}_c$. Eigenvalues of the linearization around the leukemic steady state are: $(2a_1^c - 1)p_1^c - (2a_1^l - 1)p_1^l$, $-d_2^c$, $-\bar{d}'_c \bar{l}_1$, $-d_2^l$, where $\bar{d}'_c := \frac{d}{dx} d_c(x)|_{x=\bar{l}_1+\chi\bar{l}_2+\bar{c}_1} > 0$. Therefore, all eigenvalues are negative. The instability of the purely hematopoietic steady state follows since $(2a_1^l - 1)p_1^l - d_1^l > 0$ is an eigenvalue of the corresponding linearization.

$\square$

Proposition 17 (Case $[2a_1^c - 1]p_1^c > [2a_1^l - 1]p_1^l$)**.** *Let $(2a_1^c - 1)p_1^c > (2a_1^l - 1)p_1^l$. Under the Assumptions 12 and 14 there exists a unique mixed steady state. This steady state is linearly asymptotically stable. The unique purely leukemic and the unique purely hematopoietic steady steady state are unstable.*

Proof. of Proposition 17

Existence of the mixed steady state follows from follows from Proposition 13 (iii).

We linearize around the mixed steady state. We note that $\frac{d}{dc_1}[(2a_1^c s - 1)p_1^c c_1 - d_c(c_1 + l_1)c_1]|_{c_1=\bar{c}_1, l_1=\bar{l}_1, s=\bar{s}} = -\frac{d}{dx} d_c(x)|_{x=\bar{c}_1+\bar{l}_1} \bar{c}_1$. Analogously, $\frac{d}{dl_1}[(2a_1^l - 1)p_1^l l_1 - d_c(c_1 + l_1)l_1]|_{c_1=\bar{c}_1, l_1=\bar{l}_1, s=\bar{s}} = -\frac{d}{dx} d_c(x)|_{x=\bar{c}_1+\bar{l}_1} \bar{l}_1$. We use the notation $\bar{d}' := \frac{d}{dx} d_c(x)|_{x=\bar{c}_1+\bar{l}_1}$. Furthermore, we notice $\frac{d}{dc_2} \frac{1}{1+kc_2} = \frac{-k}{(1+kc_2)^2} = -ks^2$. We obtain the following linearization:

$$\mathcal{L} = \begin{pmatrix} -\bar{d}'\bar{c}_1 & -2a_1^c p_1^c k \bar{s}^2 \bar{c}_1 & -\bar{d}'\bar{c}_1 & 0 \\ 2(1 - a_1^c \bar{s})p_1^c & 2a_1^c p_1^c k \bar{s}^2 \bar{c}_1 - d_2^c & 0 & 0 \\ -\bar{d}'\bar{l}_1 & 0 & -\bar{d}'\bar{l}_1 & 0 \\ 0 & 0 & 2(1 - a_1^l)p_1^l & -d_2^l \end{pmatrix}.$$

We immediately see that one eigenvalue is $-d_2^l$. For the remaining 3×3 matrix we simplify notation. We set:

$$\tilde{\mathcal{L}} := \begin{pmatrix} a & b & a \\ c & -b-e & 0 \\ d & 0 & d \end{pmatrix}.$$

We obtain for the characteristic polynomial $\chi_{\tilde{\mathcal{L}}}(X) = X^3 + X^2(b+e-a-d) + X(-ab-ae-bc-bd-de) + dbc$. We use the Routh-Hurwitz Criterion[23] to check, if real parts of eigenvalues have negative sign. To obtain eigenvalues, which all have negative real parts it is necessary and sufficient that

(i) $b+e-a-d>0$,

(ii) $(b+e-a-d)(-ab-ae-bc-bd-de)-dbc>0$,

(iii) $dbc>0$.

We note that (iii) is true, since $dbc = [-\bar{d}'\bar{l}_1][-2a_1^c p_1^c k\bar{s}^2\bar{c}_1][2(1-a_1^c\bar{s})p_1^c] = 4\bar{d}'\bar{l}_1(1-a_1^c\bar{s})(p_1^c)^2 a_1^c k\bar{s}^2\bar{c}_1 > 0$. We then check (i). We note that $b+e = -\frac{\xi_l+p_1^c}{2a_1^c p_1^c}\frac{\xi_c-\xi_l}{p_1^c-\xi_l}d_2^c + d_2^c$. Furthermore, $b+e>0 \Leftrightarrow \quad (p_1^c)^2 + \xi_l\xi_l - 2\xi_l\xi_c > 0$. We know that $\xi_c = (2a_1^c-1)p_1^c \le p_1^c$, therefore, $(p_1^c)^2 + \xi_l\xi_l - 2\xi_l\xi_c > \xi_c\xi_c + \xi_l\xi_l - 2\xi_l\xi_c = (\xi_l-\xi_c)^2 > 0$. Therefore, $b+e>0$, since $-a>0$ and $-d>0$ statement (i) follows.

We expand (ii):

$$\begin{aligned}
&(b+e-a-d)(-ab-ae-bc-bd-de)-dbc \\
&= \underbrace{a^2b + 2abd + bd^2 + a^2e + 2ade + d^2e}_{=:\alpha} \\
&\quad \underbrace{-ab^2 - abe - ae^2 - abe - bde - b^2d - bde - de^2}_{=:\beta} \\
&\quad \underbrace{+abc - b^2c - bce}_{=abc-bc(b+e)}
\end{aligned}$$

We obtain $\alpha = (a+d)^2(b+e) > 0$, since we have shown above that $b+e>0$. It holds $\beta = -a(b+e)^2 - d(b+e)^2 > 0$, since $-a = \bar{d}'\bar{c}_1 > 0$ and $-d = \bar{d}'\bar{l}_1 > 0$. Furthermore, $abc - bc(b+e) > 0$, since $(b+e)>0$, as shown above and it holds $-bc = -[-2a_1^c p_1^c k\bar{s}^2\bar{c}_1][2(1-a_1^c\bar{s})p_1^c] = 4(1-a_1^c\bar{s})a_1^c(p_1^c)^2 k\bar{s}^2\bar{c}_1 > 0$ and $abc = 4(1-a_1^c\bar{s})a_1^c(p_1^c)^2 k\bar{s}^2(\bar{c}_1)^2\bar{d}' > 0$. This yields (ii). We conclude that all eigenvalues have negative real parts. For the

instability of the full leukemic steady state, we check that $2(1-a_1^c)p_1^c-(2a_1^l-1)p_1^l > 0$ is an eigenvalue of the corresponding linearization. Instability of the purely hematopoietic follows since $(2a_1^l - 1)p_1^l - d_1^l > 0$ is an eigenvalue of the corresponding linearization.

□

Proposition 18 (Case $[2a_1^c - 1]p_1^c = [2a_1^l - 1]p_1^l$). *Let $(2a_1^c - 1)p_1^c = (2a_1^l - 1)p_1^l$. Let Assumptions 12 and 14 hold. Then, there exists no mixed steady state of system* (6)-(8)*. The unique purely leukemic steady state is linearly asymptotically stable. The unique purely hematopoietic steady state is unstable.*

Proof. of Proposition 18

Non-existence of the mixed steady state follows from Proposition 13 (iii). We now linearize around the leukemic steady state and obtain

$$\mathcal{L} = \begin{pmatrix} (2a_1^c - 1)p_1^c - (2a_1^l - 1)p_1^l & 0 & 0 & 0 \\ 2(1 - a_1^c)p_1^c & -d_2^c & 0 & 0 \\ -\bar{d}'\bar{l}_1 & 0 & -\bar{d}'\bar{l}_1 & 0 \\ 0 & 0 & 2(1 - a_1^l)p_1^l & -d_2^l \end{pmatrix} =: \begin{pmatrix} 0 & 0 & 0 & 0 \\ a & b & 0 & 0 \\ c & 0 & c & 0 \\ 0 & 0 & g & e \end{pmatrix}$$

We note that $b < 0, c < 0, e < 0$ are eigenvalues. Therefore, the system approaches a center manifold. We are interested in the dynamics in the vicinity of the center manifold. We apply a coordinate transformation: $\tilde{c}_1 = c_1$, $\tilde{c}_2 = \frac{a}{b}c_1 + c_2$, $\tilde{l}_1 = c_1 + l_1$ and $\tilde{l}_2 = -\frac{g}{e}c_1 + l_2$. This yields:

$$\begin{aligned} \frac{d}{dt}\tilde{c}_1 &= \left(\frac{2a_1^c}{1 + k(-a\tilde{c}_1/b + \tilde{c}_2)} - 1\right) p_1^c\tilde{c}_1 - d(\tilde{l}_1)\tilde{c}_1 \\ \frac{d}{dt}\tilde{c}_2 &= \frac{a}{b}\left(\frac{2a_1^c}{1 + k(-a\tilde{c}_1/b + \tilde{c}_2)} - 1\right) p_1^c\tilde{c}_1 - \frac{a}{b}d(\tilde{l}_1)\tilde{c}_1 \\ &\quad + 2\left(1 - \frac{a_1^c}{1 + k(-a\tilde{c}_1/b + \tilde{c}_2)}\right) p_1^c\tilde{c}_1 - d_2^c\tilde{c}_2 + \frac{a}{b}d_2^c\tilde{c}_1 \end{aligned} \tag{9}$$

$$\begin{aligned} \frac{d}{dt}\tilde{l}_1 &= \left(\frac{2a_1^c}{1 + k(-a\tilde{c}_1/b + \tilde{c}_2)} - 1\right) p_1^c\tilde{c}_1 - d(\tilde{l}_1)\tilde{c}_1 \\ &\quad + (2a_1^l - 1)p_1^l(\tilde{l}_1 - \tilde{c}_1) - d(\tilde{l}_1)(\tilde{l}_1 - \tilde{c}_1) \\ \frac{d}{dt}\tilde{l}_2 &= -\frac{g}{e}\left(\frac{2a_1^c}{1 + k(-a\tilde{c}_1/b + \tilde{c}_2)} - 1\right) p_1^c\tilde{c}_1 + \frac{g}{e}d(\tilde{l}_1)\tilde{c}_1 \\ &\quad + 2(1 - a_1^l)p_1^l(\tilde{l}_1 - \tilde{c}_1) - d_2^l(\frac{g}{e}\tilde{c}_1 + \tilde{l}_2) \end{aligned}$$

We transform the steady state to the new coordinates and obtain $\bar{\tilde{c}}_1 = 0$, $\bar{\tilde{c}}_2 = 0$, $\bar{\tilde{l}}_1 = \bar{l}_1$ and $\bar{\tilde{l}}_2 = \bar{l}_2$. We now introduce the coordinate change $\hat{c}_1 = \tilde{c}_1$, $\hat{c}_2 = \tilde{c}_2$, $\hat{l}_1 = \tilde{l}_1 - \bar{\tilde{l}}_1, \hat{l}_2 = \tilde{l}_2 - \bar{\tilde{l}}_2$. Then, it holds for the steady state $\bar{\hat{c}}_1 = \bar{\hat{c}}_2 = \bar{\hat{l}}_1 = \bar{\hat{l}}_2 = 0$.

In the new coordinates there exists a center manifold, that is tangent to $(1,0,0,0)^T$ in the origin[24]. The center manifold can be given as a graph of $\hat{c}_1$. If there exists a Taylor expansion of the center manifold, this is given by $\hat{c}_2 = q(\hat{c}_1) := \alpha\hat{c}_1^2 + \mathcal{O}(\hat{c}_1^3)$, $\hat{l}_1 = r(\hat{c}_1) := \beta\hat{c}_1^2 + \mathcal{O}(\hat{c}_1^3)$, $\hat{l}_2 = w(\hat{c}_1) := \gamma\hat{c}_1^2 + \mathcal{O}(\hat{c}_1^3)$. We now reduce the dynamic to the center manifold. We notice that time evolution of $\hat{c}_1$ is independent of $\hat{l}_2$ but dependent on $\hat{c}_2$ and $\hat{l}_1$. Therefore, we calculate α and β. On the center manifold it holds $\frac{d}{dt}\hat{c}_2 = q'(\hat{c}_1)\frac{d}{dt}\hat{c}_1$, $\frac{d}{dt}\hat{l}_1 = r'(\hat{c}_1)\frac{d}{dt}\hat{c}_1$. We expand the ODE for $\hat{c}_1$ near the center manifold and obtain: $\frac{d}{dt}\hat{c}_1 = 2a_1^c p_1^c k\frac{a}{b}\hat{c}_1^2 + \mathcal{O}(\hat{c}_1^3)$. We, therefore, obtain $q'(\hat{c}_1)\frac{d}{dt}\hat{c}_1 = \mathcal{O}(\hat{c}_1^3)$ and $r'(\hat{c}_1)\frac{d}{dt}\hat{c}_1 = \mathcal{O}(\hat{c}_1^3)$. We reduce the ODE for $\hat{c}_2$ to the center manifold and obtain: $\frac{d}{dt}\hat{c}_2 = 2a_1^c p_1^c k\frac{a}{b}\hat{c}_1^2\left(\frac{a}{b}-1\right) - d_2^c\alpha\hat{c}_1^2 + \mathcal{O}(\hat{c}_1^3)$.

For $\alpha = \frac{2a_1^c p_1^c k\frac{a}{b}\left(\frac{a}{b}-1\right)}{d_2}$ it holds $q'(\hat{c}_1)\frac{d}{dt}\hat{c}_1 = \frac{a}{b}\left(\frac{2a_1^c}{1+k(-a\hat{c}_1/b+\hat{c}_2)} - 1\right)p_1^c\hat{c}_1 - \frac{a}{b}d(\hat{l}_1 + \bar{l}_1)\hat{c}_1 + 2\left(1 - \frac{a_1^c}{1+k(-a\hat{c}_1/b+\hat{c}_2)}\right)p_1^c\hat{c}_1 - d_2^c\hat{c}_2 + \frac{a}{b}d_2^c\hat{c}_1$, up to terms of order $\mathcal{O}(\hat{c}_1^3)$. For the dynamics of $\hat{l}_1$ on the center manifold we obtain: $\frac{d}{dt}\hat{l}_1 = 2a_1^c p_1^c\frac{a}{b}k\hat{c}_1^2 - d'(\bar{l}_1)\bar{l}_1\beta c_1^2 + \mathcal{O}(\hat{c}_1^3)$. For $\beta = \frac{2a_1^c p_1^c\frac{a}{b}k}{d'(\bar{l}_1)\bar{l}_1}$ it results $r'(\hat{c}_1)\frac{d}{dt}\hat{c}_1 = \left(\frac{2a_1^c}{1+k(-a\hat{c}_1/b+\hat{c}_2)} - 1\right)p_1^c\hat{c}_1 - d(\hat{l}_1+\bar{l}_1)\hat{c}_1 + (2a_1^l-1)p_1^l(\hat{l}_1+\bar{l}_1-\hat{c}_1) - d(\hat{l}_1+\bar{l}_1)(\hat{l}_1+\bar{l}_1-\hat{c}_1)$, up to order $\mathcal{O}(\hat{c}_1^3)$. Insertion of the expansions into equation (9) and Taylor expansion yields $\frac{d}{dt}\hat{c}_1 = 2a_1^c p_1^c k\frac{a}{b}\hat{c}_1^2 + \mathcal{O}(\hat{c}_1^3)$. Since $2a_1^c p_1^c k\frac{a}{b} < 0$, $\hat{c}_1$ converges to zero and also $\hat{c}_2$, $\hat{l}_1$, $\hat{l}_2$ for $t \to \infty$.

□

Remark 19. *Propositions 16-18 suggest that the mixed steady state is the only linearly stable state whenever it exists.*

2.3 Leukemic stem cell properties

In this section, we ask how leukemic stem cells are characterized in Model 2. We define that l_1 is the leukemic stem cell population of system (3) - (5) if it can maintain a steady state of leukemic cells. This means that there exists a steady state of system (3) - (5) with $\bar{l}_1 > 0$. This definition is analogous to the definition of HSC in[4].

Proposition 20. *Let Assumptions 2 be fulfilled. Consider a non-negative steady state* $(\bar{c}_1, \ldots, \bar{c}_n, \bar{l}_1, \ldots, \bar{l}_m)$ *of system* (3)-(5).

(i) Define

$$j := \max\{i | (2a_i^l - 1)p_i^l - d_i^l \geq (2a_k^l - 1)p_k^l - d_k^l \text{ for all } k\}.$$

Then, $\bar{l}_i = 0$ *for all* $1 \leq i < j$.

(ii) If $(2a_j^l - 1)p_j^l - d_j^l > 0$ *for* j *defined as in (i), then there exists a purely leukemic steady state with* $\bar{l}_j > 0$ *and* $\bar{l}_i = 0$ *for* $i < j$.

Proof. (i) Let $j > 1$, otherwise the statement is trivial. Let $\bar{l}_k > 0$, $k < j$ and k chosen minimal. Then $\frac{d}{dt} l_k = 0$ implies that $\bar{d}_c = (2a_k^l - 1)p_k^l - d_k^l$. Since $2(1 - a_i^l) > 0$ for all i, it follows inductively that $\bar{l}_m > 0$ for $m > k$. Then it follows that $\frac{d}{dt} l_j > 0$, since $2(1 - a_{j-1}^l)p_{j-1}^l \bar{l}_{j-1} > 0$ and $(2a_j^l - 1)p_j^l - d_j^l - \bar{d}_c \geq 0$ due to the definition of j. This is a contradiction, since $\frac{d}{dt} l_j$ has to be zero in steady state. (ii) follows from an explicit calculation. □

Remark 21. *It follows from Proposition 20 that the leukemic stem cell population is characterized by maximality of* $(2a_i^l - 1)p_i^l - d_i^l$. *To obtain steady states with* $l_1 > 0$, *we therefore have to assume* $\big((2a_1^l - 1)p_1^l - d_1^l\big) - (2a_i^l - 1)p_i^l + d_i^l > 0$ *for all* $i > 1$.

2.4 Criteria for instability of steady states

In this section we derive criteria for instability of steady states of system (3) - (5). These criteria are biologically meaningful, since expansion of leukemic cells can be interpreted as destabilization of the healthy equilibrium. Vice versa treatment strategies correspond to re-stabilization of healthy states.

Proposition 22. *Let Assumptions 2 be fulfilled. Assume there exists a steady state* $(0, \ldots, 0, \bar{c}_k, \ldots \bar{c}_n, 0, \ldots, 0, \bar{l}_j, \ldots, \bar{l}_m)$ *of system* (3) - (5) *with* $\bar{c}_k > 0$ *and* $\bar{l}_j > 0$ *then*

(i) each steady state with $\bar{l}_j = 0$ *is unstable.*

(ii) each steady state with $\bar{c}_k = 0$ *and* $\bar{l}_{j-1} = 0$ *and* $\bar{l}_j > 0$ *is unstable,*

For $k = 1$ *or* $j = 1$ *we consider the condition* $\bar{c}_{k-1} = 0$ *or* $\bar{c}_{j-1} = 0$ *to be trivially fulfilled.*

Proof. The proof follows from linearization. □

Remark 23. *Due to Proposition 22 all steady states, which do not have the maximal number of positive leukemic populations, are unstable. This implies that if there exists a steady state with positive leukemic cell numbers, i.e.,* $(2a_i^l - 1)p_i^l - d_i^l > 0$ *for at least one* i*, the healthy equilibrium is unstable.*

2.5 Comparison of Models 1 and 2

In this section, we sketch properties of Model 1 and compare them to Model 2. A detailed analysis of Model 1 can be found in[18]. Although qualitative behavior of both models is similar for wide ranges of parameters, there exist differences that may be helpful to discriminate between them. Results are summarized in Table 1.

2.5.1 Steady states

For suitable parameters both models can have purely leukemic, purely hematopoietic and mixed steady states. In Model 1 mixed steady states are not unique and they form a one-dimensional manifold. The condition for existence of mixed steady states in case of Model 1 is $\frac{a_1^l p_1^l}{d_1^l + p_1^l} = \frac{a_1^c p_1^c}{d_1^c + p_1^c}$ [18]. Coexistence is an improbable event, since the subset of parameters compatible with coexistence have Lebesgue-measure zero in parameter space. In Model 2 mixed steady states are unique and coexistence is a more probable event, since the subset of parameters suitable for coexistence has positive measure. Coexistence can occur if and only if $0 < (2a_1^l - 1)p_1^l - d_1^l < (2a_1^c - 1)p_1^c - d_1^c$, see Proposition 13. In this case feedback stimulation prevents decline of healthy cells.

2.5.2 Leukemic stem cell properties

In Model 2 the leukemic stem cell population is characterized by $(2a_{LSC} - 1)p_{LSC} - d_{LSC} \geq (2a_i^l - 1)p_i^l - d_i^l$ for all i, see Remark 21 (i). The condition for destabilization of the healthy state is $(2a_{LSC} - 1)p_{LSC} - d_{LSC} > 0$, see Remark 23. In Model 1 the leukemic stem cell population is characterized by $\frac{a_{LSC} p_{LSC}}{d_{LSC} + p_{LSC}} \geq \frac{a_i^l p_i^l}{d_i^l + p_i^l}$ for all i and the criterion for destabilization of the healthy state is $\frac{a_{LSC} p_{LSC}}{d_{LSC} + p_{LSC}} > \frac{a_{HSC} p_{HSC}}{d_{HSC} + p_{HSC}}$, see[18]. If we assume that leukemic stem cells do not die if marrow is not over-crowded the conditions simplify to $a_{LSC} > a_{HSC}$ in case of Model 1 and to $a_{LSC} > 0.5$ in case of Model 2. We note that in Model 1 the relation between HSC and LSC parameters decides if a leukemic cell expands, whereas in Model 2 the absolute value of self-renewal matters. This is plausible, since in Model 1 healthy

and malignant cells depend on identical feedback signals. To gain a competitive advantage, leukemic cells need to respond to environmental signals more efficiently than their benign counterparts. In Model 2 the competitive advantage of malignant cells is their independence from environmental signals.

2.5.3 Stability results

Results on linearized stability refer to the case $m = n = 2$ and $d_1^l = d_1^c = 0$. In Model 1 the purely hematopoietic steady state is linearly asymptotically stable if and only if $a_1^l < a_1^c$. In this case the purely leukemic steady state is unstable. The purely leukemic steady state is linearly asymptotically stable if and only if $a_1^l > a_1^c$, then the purely hematopoietic steady state is unstable[18]. Mixed steady states occur in the case $a_1^l = a_1^c$. The mixed steady states of Model 1 form a one dimensional manifold which is a center manifold. The purely leukemic and the healthy steady state are elements of it. Depending on parameters, this manifold can either be attractive or repulsive, meaning linear stability or instability[18]. In Model 2, the purely hematopoietic steady state is linearly asymptotically stable if and only if $a_1^l < 0.5$. Unlike in Model 1 this condition is independent of hematopoietic cell properties. The purely leukemic steady state is linearly asymptotically stable if and only if $(2a_1^l - 1)p_1^l \geq (2a_1^c - 1)p_1^c > 0$ (Propositions 16 and 18), then the purely hematopoietic steady state is unstable. Unlike in Model 1 the mixed steady states of Model 2 are always linearly asymptotically stable, see Proposition 17. A common feature of both models is instability of steady states with less than the maximal number of positive components (Remark 23).

3 Simulation and Fitting

3.1 Simulation of cytokine administration

We simulate cytokine administration by setting $s = 1$ for the duration of external cytokine supplementation. In Model 1 the cytokine feedback mechanism prevents unbounded expansion of cells in absence of external cytokines. However, when we set $s = 1$, we would observe unbounded cell growth in Model 1. To take into account crowding of the marrow space, we include in Model 1 increased death rates due to marrow crowding, as in Model 2, i.e., for $1 \leq i < n$ we add $-d_c(t)c_i = -\hat{d}_c(\sum_{k=1}^{n-1} c_k + \sum_{k=1}^{m-1} l_k)c_i$ to the ODE describing time evolution of c_i and the same for l_i. The function $\hat{d}_c$ fulfills

	**Cytokine-dependent Leukemia (Model 1)**	**Cytokine-independent Leukemia (Model 2)**
**Coexistence**	rare, always manifold of steady states, stable or unstable	more probable than in Model 1, unique steady states, for $m = n = 2$ always stable
**Leukemia expansion**	slower than in Model 2	faster than in Model 1
**Destabilization by LSC ($d_1^l = 0$)**	LSC self-renewal > HSC self-renewal	LSC self-renewal > 0.5
**LSC characterization**	$\frac{a_{LSC} p_{LSC}}{d_{LSC}+p_{LSC}} \geq \frac{a_i^l p_i^l}{d_i^l+p_i^l}$ for all i, $(2a_{LSC}-1)p_{LSC}-d_{LSC} > 0$	$(2a_{LSC}-1)p_{LSC}-d_{LSC} \geq (2a_i^l-1)p_i^l - d_i^l$ for all i, $(2a_{LSC}-1)p_{LSC}-d_{LSC} > 0$

Supplemental Table 1: **Comparison of Models**

Assumption 6 (iv). If $\hat{x}$ from Assumption 6 (iv) is chosen large enough, e.g., twice the steady state marrow cell count, the function d_c does not affect dynamics of Model 1 in absence of cytokine stimulation, since the feedback signal limits total cell counts.

3.2 Fitting to data of 41 patients

To fit the model to data of the 41 patients collected at Heidelberg University, we use the 2+2 compartment versions of Models 1 and 2 (2 hematopoietic and 2 leukemic compartments, $n = m = 2$). We assume that c_1, l_1 are located in marrow and c_2, l_2 are located in blood stream, i.e., $\chi = 0$. To convert cell counts per kg of body weight to cell counts per liter of blood, we assume a body weight of 80 kg and a blood volume of 5 liters.

Healthy cell parameters were taken from previous work[25], i.e., $a_1^c = 0.87$, $p_1^c = 0.45/day$, $k^c = 1.85 \cdot 10^{-9} kg$, $d_1^c = 0$, $d_2^c = 2.3/day$. Healthy cell parameters, i.e., a_1^c, p_1^c, k^c, d_1^c, d_2^c, are assumed to be the same for all patients. Leukemic cell parameters are considered to be patient specific. The patient specific parameters in Model 1 are a_1^l, p_1^l, d_2^l, k^l. The patient specific parameters in Model 2 are a_1^l, p_1^l, d_2^l, $\hat{x}$, $\hat{d}$. For each individual patient we use a least-square fitting approach to identify leukemic cell parameters that optimally reproduce the observed blast dynamics. The purpose of the parameter fitting is to determine which of the two models are able to capture the individual dynamics of a given patient. During the fitting we restrict

parameter values to biologically plausible ranges:

- Leukemic cell proliferation rates are varied between one division per two years ($p_1^l = 9 \cdot 10^{-4}$/day) and one division per day ($p_1^l = 0.7$/day). The latter is considered as an upper bound taking into account the genome replication time of an eukaryote cell[26].

- Self-renewal can vary between zero and one, which is the natural range for a probability.

- Blast half-life is chosen between 5% and 100% of leukocyte half-life ($d_2^l \in [0.1/day; 2.3/day]$), motivated by literature[27].

- In case of Model 1 the feedback constant k^l is varied between 10% and 1000% of the value for the healthy cell feedback constant k^c, i.e., $k^l \in [1.85 \cdot 10^{-10} kg, 1.85 \cdot 10^{-8} kg]$, which is based on previous modeling results[25].

- The function d_c in Model 2 has the following form: $d_c(x) = \hat{d} \max(x - \hat{x})$, as in[25]. The value $\hat{x}$ is varied between 150% and 300% of the healthy marrow cell count, i.e., $\hat{x} \in [3 \cdot 10^9/kg, 6 \cdot 10^9/kg]$ (in the healthy system $\hat{x}$ is considered to be 200% of steady state marrow cell count[25]). The value of $\hat{d}$ is varied between $10^{-13}/day$ and $10^{-9}/day$, in accordance with previous studies[25].

- To fit the models to the data of relapsing patients we start computer simulations with equilibrium cell counts in the hematopoietic lineage and a small number of LSC (1 per kg of body weight), mimicking the appearance of LSC due to a mutation or survival of LSC after therapy.

The fitted self-renewal fractions are distributed uniformly in $(a_1^c, 1)$ for Model 1 and in $(0.5, 1)$ for Model 2. All other fitted parameters are uniformly distributed in their biologically plausible ranges defined above.

The patients shown in Figure 4 have relatively high blast counts at relapse. In these patients, dynamics of both fitted models look very similar to each other during time intervals when blast counts are low. This however does not imply that our approach is limited to patients with high blast counts. When fitting both models to patients with low blast counts (blasts $\leq$ 20%) differences in the dynamics of the fitted models become evident also for low blast counts. Supplemental Figure 2 shows examples for such patients.

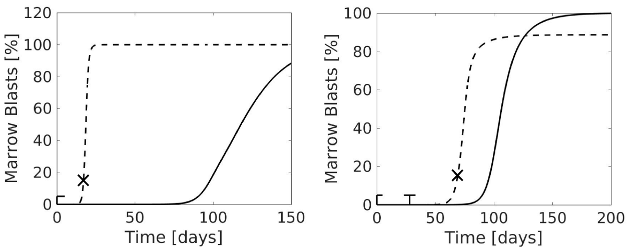

Supplemental Figure 2: Examples for patients with low blast counts. The models of cytokine-dependent (solid line) and cytokine-independent (dotted line) AML have been fitted to patient data with low blast counts at relapse. In all three examples the model fits suggest that the AMLs are cytokine-independent. Bone marrow blast fractions are marked by "x" if exact values were reported, if intervals have been reported, the data is shown as a bar.

3.3 Fitting to data of cytokine administration

As a proof of principle, we also fitted the model to the data from[28], showing a blast crisis in response to administration of pegfilgrastim. The data includes peripheral blast counts and marrow blast data. After induction therapy 51% marrow blast are reported, which we take as initial condition for our simulations. The parameters of the healthy system are chosen as reported in Section 3.2 of this Supplement. The absolute values of c_1 and l_1 at the beginning of the simulation are left as a free parameter, they are varied between 1% and 90% of the healthy steady state value for $\bar{c}_1$, i.e, marrow cellularity varies between 2% and 180% of healthy marrow. All other parameters were varied as described above. During intervals of cytokine stimulation, we set $s = 1$. We assume that, as after the first therapy cycle, the marrow blast percentage is around 50% after the second therapy cycle. We neglect the last two pre-mortal peripheral blast measurements, since they show a decline of the periperal blast count which can have multiple reasons, that are not covered by the model, e.g., increase of plasma volume due to renal failure.

3.4 Simulations shown in Figure 3

To obtain the simulation in Figure 3 we chose healthy cell parameters as described above ($a_1^c = 0.87$, $p_1^c = 0.45/day$, $d_2^c = 2.3/day$, $d_1^c = d_1^l = 0$, $k^c = 1.85 \cdot 10^{-9}$). For the leukemic cells we set $d_2^l = 0.1/day$, based on measurements of apoptosis in leukemic blasts[27,29]. LSC proliferation p_1^l is

set to 0.7, corresponding to one division per day. To take into account cell death we set $d_c(x) = \max(0, x - 4 \cdot 10^9) \cdot 10^{-10}$, $\chi = 0$. This implies that if the marrow cell count exceeds a given threshold, the death rate increases linearly with marrow cell counts. Bone marrow histology suggests that around 50% of bone marrow consist of fat that can be replaced by hematopoietic tissue[19]. Therefore, the additional death rate d is zero as long as marrow cell count is below twice the steady state marrow cell count ($4 \cdot 10^9$ cells per kg). The choice of d_c implies that if bone marrow cell counts are three times the steady state cell counts, the additional death rate due to overcrowding is of the order of magnitude of mature cell clearance. Simulations with other parameters for d_c result in the same qualitiative behavior. If we measure the time until decrease of healthy mature cell counts a similar result is obtained.

3.5 Simulations shown in Figure 6

The examples in Figure 6 have been obtained using the following parameters, which have been justified in Section 3.2 of this Supplement: $a_1^c = 0.87$, $p_1^c = 0.45$, $k^c = 1.85 \cdot 10^{-9}$, $d_2^c = 2.3$, $d_2^l = 0.1$, $\hat{d_c}(x) = \max(0, x - 5 \cdot 10^9) 10^{-9}$, $k^c = k^l = k$. Panels A and B consider the model of cytokine-dependent AML. Panel A shows an example for the case $a_1^l > a_1^c$ and $(2a_1 - 1)p_1 > (2a_1^l - 1)p_1^l$, we set $a_1^l = 0.875$, $p_1^l = 0.2$. Panel B shows an example for the case $a_1^l > a_1^c$ and $(2a_1^c - 1)p_1^c < (2a_1^l - 1)p_1^l$, we set $a_1^l = 0.9$, $p_1^l = 0.8$. Panels C and D consider the model of cytokine-independent AML. In panel C we provide an example for the case $(2a_1^l - 1)p_1^l > (2a_1^c - 1)p_1^c$, we set $a_1^l = 0.8$, $p_1^l = 0.7$ or $a_1^l = 0.9$ $p_1^l = 0.525$ (numerical results are identical for both cases, since system dynamics depend only on the value of $(2a_1^l - 1)p_1^l$). In panel D we consider the opposite case $(2a_1^c - 1)p_1^c < (2a_1^l - 1)p_1^l$ and set $a_1^l = 0.8$, $p_1^l = 0.465$ or $a_1^l = 0.965$, $p_1^l = 0.3$. Cytokine administration is simulated as described in Section 3.1. (numerical results are identical for both cases). All parameters from the given examples are within the parameter ranges obtained from fitting of patient data. All parameters are listed in Supplemental Table 2. Supplemental Figure 3 shows the content of Figure 6 using linear scales.

**Panels**	**Parameters**
A-D	$a_1^c = 0.87$<br>$p_1^c = 0.45/day$<br>$k^c = 1.85 \cdot 10^{-9} kg$<br>$d_2^c = 2.3/day$<br>$d_2^l = 0.1/day$<br>$\hat{d}_c(x) = \max(0, x - 5 \cdot 10^9) \cdot 10^{-9}/day$<br>(corresponding to a maximal effective growth rate of $0.333/day$)
A	$a_1^l = 0.875$<br>$p_1^l = 0.2/day$<br>$k^l = k^c$<br>(corresponding to a maximal effective growth rate of $0.15/day$)
B	$a_1^l = 0.9$<br>$p_1^l = 0.8/day$<br>$k^l = k^c$<br>(corresponding to a maximal effective growth rate of $0.64/day$)
C	$a_1^l = 0.9$<br>$p_1^l = 0.525/day$<br>(corresponding to an effective growth rate of $0.42/day$)
D	$a_1^l = 0.965$<br>$p_1^l = 0.3/day$<br>(corresponding to an effective growth rate of $0.279/day$)

Supplemental Table 2: Parameters used for simulations shown in Figure 6

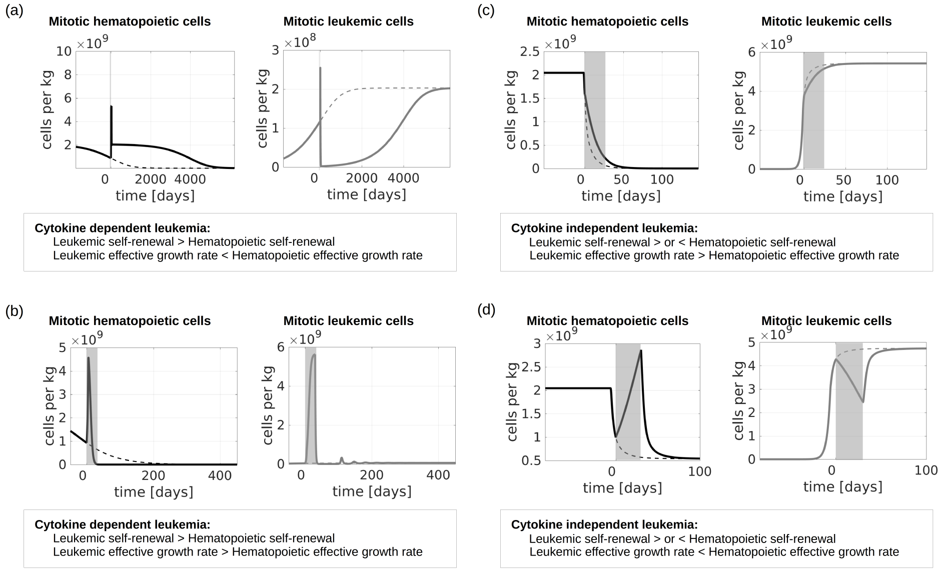

Supplemental Figure 3: Effect of cytokine stimulation on leukemic cell burden. This Figure shows the same content as Figure 6 of the main text. The only difference is that in this Figure linear scales are used. When mature cell counts are reduced by 50% due to leukemic cell load we simulate cytokine stimulation for 30 days. Dotted lines show dynamics in absence of cytokine stimulation, solid lines show dynamics during and after cytokine stimulation. (A) Cytokine dependent AML, the self-renewal of leukemic cells is higher than the self-renewal of hematopoietic cells and the effective growth rate of leukemic cells is smaller than the effective growth rate of hematopoietic cells. Cytokine administration reduces the leukemic cell burden. (B) Cytokine dependent AML, the self-renewal of leukemic cells is higher than the self-renewal of hematopoietic cells and the effective growth rate of leukemic cells is higher than the effective growth rate of hematopoietic cells. Cytokine administration increases the leukemic cell burden. (C) Cytokine independent AML, the effective growth rate of leukemic cells is larger than the effective growth rate of hematopoietic cells, leukemic cell self-renewal can be larger or smaller than hematopoietic cell self-renewal. Cytokines cannot reduce the leukemic cell burden, but they slightly reduce leukemic cell expansion. (D) Cytokine independent AML, the effective growth rate of leukemic cells is smaller than the effective growth rate of hematopoietic cells, leukemic cell self-renewal can be larger or smaller than hematopoietic cell self-renewal. In this case leukemic cell counts can be reduced by cytokine administration. Simulation details are provided in the Supplement (Section 3.5). All parameters are listed in Supplemental Table 2.

References

1. Jandl JH, Blood cell formation. In: Jandl JH, ed., Textbook of Hematology, Boston, MA: Little, Brown and Company, 1996;1–69.

2. Smith C, Production, distribution and fate of neutrophils. In: Kaushansky K, Lichtman M, Beutler E, Kipps T, Seligsohn U, Prchal J, eds., Williams Hematology, 8th ed., New York: Mc Graw Hill, 2010;891–896.

3. Marciniak-Czochra A, Stiehl T, Jäger W, Ho AD, Wagner W, Modeling of asymmetric cell division in hematopoietic stem cells – regulation of self-renewal is essential for efficient repopulation. *Stem Cells Dev.* 2009; 18:377–385.

4. Stiehl T, Marciniak-Czochra A, Characterization of stem cells using mathematical models of multistage cell lineages. *Mathematical and Computer Modelling* 2011;53:1505–1517.

5. Stiehl T, Ho A, Marciniak-Czochra A, The impact of CD34+ cell dose on engraftment after SCTs: personalized estimates based on mathematical modeling. *Bone Marrow Transplant* 2014;49:30–7.

6. Stiehl T, Ho A, Marciniak-Czochra A, Assessing hematopoietic (stem-) cell behavior during regenerative pressure. *Adv Exp Med Biol.* 2014; 844:347–367.

7. Marciniak-Czochra A, Stiehl T, Mathematical models of hematopoietic reconstitution after stem cell transplantation. In: Bock H, Carraro T, Jaeger W, Koerkel S, eds., Model Based Parameter Estimation: Theory and Applications., Heidelberg, Springer, 2011;.

8. Stiehl T, Baran N, Ho A, Marciniak-Czochra A, Cell division patterns in acute myeloid leukemia stem-like cells determine clinical course: a model to predict patient survival. *Cancer Res* 2015;75:940–949.

9. Marciniak-Czochra A, Lutz C, Stiehl T, Emergence of heterogeneity in acute leukemias. *Biology Direct* 2016;11:51.

10. Stiehl T, Marciniak-Czochra A, Stem cell self-renewal in regeneration and cancer: Insights from mathematical modeling. *Curr Opin Systems Biology* 2017;5:112–120.

11. Metcalf D, Hematopoietic cytokines. *Blood* 2008;111:485–491.

12. Shier L, Schultz K, Imren S, et al., Differential effects of granulocyte colony-stimulating factor on marrow- and blood-derived hematopoietic and immune cell populations in healthy human donors. *Biol Blood Marrow Transplant.* 2004;10(9):624–34.

13. Dicke K, Hood D, Arneson M, et al., Effects of short-term in vivo administration of g-csf on bone marrow prior to harvesting. *Exp Hematol.* 1997;25(1):34–8.

14. Tegg E, Tuck D, Lowenthal R, Marsden K, The effect of g-csf on the composition of human bone marrow. *Clin Lab Haematol.* 1999;21(4):265–70.

15. MacHida U, Tojo A, Takahashi S, et al., The effect of granulocyte colony-stimulating factor administration in healthy donors before bone marrow harvesting. *Br J Hematol.* 2000;108(4):747–53.

16. Shinjo K, Takeshita A, Ohnishi K, Ohno R, Granulocyte colony-stimulating factor receptor at various stages of normal and leukemic hematopoietic cells. *Leuk Lymphoma* 1997;25:37–46.

17. Kondo S, Okamura S, Asano Y, Harada M, Niho Y, Human granulocyte colony-stimulating factor receptors in acute myelogenous leukemia. *Eur J Hematol* 1991;46:223–230.

18. Stiehl T, Marciniak-Czochra A, Mathematical modelling of leukemogenesis and cancer stem cell dynamics. *Math. Mod. Natural Phenomena.* 2012;7:166–202.

19. Loeffler H, Rastetter J, Haferlach T, Atlas of Clinical Hematology. Heidelberg: Springer, 2000, p. 78.

20. Dommange F, Cartron G, Espanel C, et al., CXCL12 polymorphism and malignant cell dissemination/tissue infiltration in acute myeloid leukemia. *FASEB J* 2006;20:1913–1915.

21. Berger M, Motta C, Boiret N, Aublet-Cuvelier B, Bonhomme J, Travade P, Membrane fluidity and adherence to extracellular matrix components are related to blast cell count in acute myeloid leukemia. *Leuk Lymphoma* 1994;15:297–302.

22. Tavor S, Petit I, Porozov S, et al., Motility, proliferation, and egress to the circulation of human AML cells are elastase dependent in NOD/SCID chimeric mice. *Blood* 2005;106:2120–2127.

23. Gantmacher F, The theory of matrices 2. Chelsea Publishing, 1964.

24. Carr J, Applications of Center Manifold Theory. Heidelberg: Springer, 1981.

25. Stiehl T, Baran N, Ho A, Marciniak-Czochra A, Clonal selection and therapy resistance in acute leukaemias: mathematical modelling explains different proliferation patterns at diagnosis and relapse. *J. R. Soc. Interface* 2014;11:20140079.

26. Morgan D, Desai A, Edgar B, et al., The Cell Cycle. In: B. Alberts, A. Johnson, J. Lewis, M. Raff, K. Roberts, R. Walter (Eds): Molecular Biology of the Cell, 5th Edition. Garland Science, 2007.

27. Savitskiy V, Shman T, Potapnev M, Comparative measurement of spontaneous apoptosis in pediatric acute leukemia by different techniques. *Cytometry B Clin Cytom* 2003;56:16–22.

28. Duval C, Boucher S, Moulin J, et al., Fatal stimulation of acute myeloid leukemia blasts by pegfilgrastim. *Anticancer Res.* 2014;34(11):6747–6748.

29. Malinowska I, Stelmaszczyk-Emmel A, Wasik M, Rokicka-Milewska R, Apoptosis and ph of blasts in acute childhood leukemia. *Med Sci Monit* 2002;8:CR441–CR447.
